# Comparative Analysis of the Protein Composition of Goat Milk from French Alpine, Nubian, and Creole Breeds and Holstein Friesian Cow Milk: Implications for Early Infant Nutrition

**DOI:** 10.3390/ani12172236

**Published:** 2022-08-30

**Authors:** Florencia Muñoz-Salinas, Héctor Mario Andrade-Montemayor, Karina De la Torre-Carbot, Miguel Ángel Duarte-Vázquez, Juan Carlos Silva-Jarquin

**Affiliations:** 1 Facultad de Ingeniería, Campus Amazcala, Universidad Autónoma de Querétaro, El Marqués, CP 76265, Mexico; 2 Facultad de Ciencias Naturales, Campus Juriquilla, Universidad Autónoma de Querétaro, Juriquilla, CP 76230, Mexico; 3 Departamento de Investigación Científica, Migh International, Carretera Nacional Jiquilpan-Manzanillo Km 39, San José de Gracia, CP 59500, Mexico; 4 Facultad de Ciencias Naturales, Campus Amazcala, Universidad Autónoma de Querétaro, El Marqués, CP 76265, Mexico

**Keywords:** goat milk proteins, cow milk proteins, caseins, alpha-lactoalbumin, infant nutrition

## Abstract

**Simple Summary:**

Goat’s milk is a food that contains proteins of value for nutrition. The protein profile in the milk of goat breeds is different from that of cow milk, with a lower relative abundance of allergenic proteins. In addition, regardless of the breed, goat milk has beta-casein of type A2 in a more significant proportion than cow milk, which impacts different bioactive peptides hydrolyzed in the milk of the species.

**Abstract:**

Of the diversity of proteins and high digestibility, goat milk will be a food of significant value for infant nutrition. The genetic polymorphisms of milk proteins play an essential role in the different degrees of allergic reactions. This work aimed to identify the proteins and peptides in the composition of goat milk and compare them to those in cow’s milk. The work was performed with goats French Alpine, Nubian, and Creole breeds and Holstein Friesian milking cows at the Universidad Autónoma de Querétaro, Amazcala. We investigated the relative abundance of goat and cow milk protein fractions by SDS-PAGE resolution and the densitometric analysis of gels. The protein alfa-casein was (17.67 ± 0.46) for Creole, (19.18 ± 0.88) French Alpine, (17.35 ± 0.49) Nubian, and (35.92 ± 1.96) Holstein cows. The relative abundance obtained from alfa-casein was statistically different between goats and cows, and this protein was vital because it is a protein related to allergies. On the other hand, the amino acid in position 67 of the beta-casein from three goat breeds is a Proline, so it is assumed that the beta-casein variant of goat milk is an A2-type. The latter has excellent relevance for infant nutrition and differs from cow milk.

## 1. Introduction

Protein supply is essential to early infant nutrition, supporting appropriate growth and development [1] and determining long-term health outcomes [2]. Human milk (HM) is an excellent source of protein and the preferred mode of nutrition for infants [3]. HM contains 0.9 ± 0.2 g/dL of total protein [4], divided mainly into caseins and whey proteins with a ratio of approximately 35:65 in mature milk [5]. HM whey protein fraction comprises a relatively high concentration of lactoferrin (LF), alpha-lactalbumin (a-LA), immunoglobulins (Ig) A, G, and M, and lysozyme C (LZC) [5]. HM caseins are mainly composed of beta-casein (BC) and kappa-casein (KC) and, to a lesser extent, alpha-casein (AC) [6].

Cow milk whey protein contains elevated concentrations of beta-lactoglobulin (b-LG) but has relatively poor levels of LF, a-LA, Ig, and LZC [7]. AC is the most abundant protein in cow milk caseins, followed by BC and KC [7]. B-LG is not expressed in HM [8], and AC is usually considered absent or present at low concentrations [6]. Furthermore, bovine BC is frequently present in cow milk as the genetic variants BC A1 and BC A2 [9]. The exchange of proline 67 in BC A2 to histidine in the same amino acid position in BC A1 facilitates the release of a seven-amino-acid peptide called beta-casomorphin 7 (BCM7, Tyr60-Ile66) during gastrointestinal digestion. BCM7 alters gastrointestinal motility in children and adults and induces apnea in infants [9].

Goat milk has a casein-to-whey protein ratio of 78:22 but a higher a-LA-to-b-LG percentage characterizes this whey protein compared to whey protein in cow milk [10]. Similar levels of LF, Ig, and LZC have been reported for goat and cow milk [11]. In addition, the casein fraction in goat milk shows higher amounts of BC and KC relative to AC [11] than in cow milk [7]. Goat milk with lower AC has a finer, softer curd [12], which may alter the physicochemical properties of casein gels formed in the stomach from goat-milk-based IF (infant formula), making them more easily digested than casein gels formed from standard IF [13], leading to faster gastric emptying [10]. Lower levels of AC and b-LG, together with speedier digestion of both proteins, may also contribute to a reduced allergenic potential for goat milk [10,14].

There is a current effort to improve the protein composition of standard IF to make the performance of formula-fed infants more similar to that of breastfed infants [15]. Goat milk has accumulated a large body of scientific and clinical evidence leading the European Food Safety Authority to conclude in 2012 that it was suitable as a source of protein and bioactive peptides for infant nutrition [16].

This work aimed to analyze the protein fraction of the milk from French Alpine, Nubian, and Creole breeds compared to that of the milk from Holstein Friesian cows. In addition, the BC fraction from goat and cow milk was sequenced by high-performance liquid chromatography coupled to the quadruple mass spectrometer (HPLC-MS/MS) to identify amino acid residue at position 67 as an indicator for the release of bioactive peptides such as BCM7.

## 2. Materials and Methods

We obtained the materials from Sigma-Aldrich (Saint Louis, MO, USA): bovine BC, a-LA, b-LG (purity ≥ 98%) and IgG (≥95%) protein standards, human milk LF protein standard (>95%), 1,4-dithiothreitol (DTT), iodoacetamide, acetonitrile, formic acid, and thermolysin (Type X, E.C. No. 3.4.24.27). Tris base, ethylenediaminetetraacetic acid (EDTA), sodium dodecyl sulfate (SDS), ammonium persulfate, N, N, N, N’-tetramethylethylenediamine (TEMED), Precision Plus Protein^TM^ standards, and colloidal Coomassie G-250 stain, all molecular biology grade, were acquired from Bio-Rad (Hercules, CA, USA). Acrylamide, N, N’-methylenebisacrylamide, urea, 2-mercaptoethanol, glycine and bromophenol blue, and ultrapure Bioreagent grade, as well as isobutyl alcohol, glacial acetic acid, and sodium hydroxide, were purchased from Thermo Fisher Scientific (Waltham, MA, USA). Thermo Fisher Scientific provided Invitrogen’s Qubit^®^ Protein Assay kit. Zip Tips C18 were obtained from Millipore Sigma (Saint Louis, MO, USA). Deionized water, used in all experiments, was purified using a Milli-Q system from Millipore Merck (Darmstadt, Germany).

### 2.1. Study Design

We worked the livestock area at the Amazcala Campus of the Autonomous University of Querétaro (UAQ) Natural Science Faculty, México. This work was authorized by the Bioethics Committee of the UAQ’s School of Natural Science Faculty (FCN) with Code No. 97FCN2014.

We performed the work with 30 milking goats, 10 of which were of the French Alpine breed, 10 of the Nubian breed, and the remaining 10 of the Creole breed. The goats were in the last third of lactation; the animals had 2 to 3 births with 1.5 to 2 L of milk produced. The number of samples was 3 per animal. The total was 30 samples for the day. We milked the goats once daily (7 to 9 a.m.). After cleaning the udders, a mobile milker (J. Delgado FLACO^TM^ brand) was used to extract the milk. Milk samples were collected and stored in a freezer at −20 °C until analysis.

### 2.2. Animal Feeding

The feeding consisted of 8 h of grazing in the semiarid range. We supplemented with 1 kg of concentrate per day (with corn grain meal, soybean meal, wheat bran, gluten meal, dry distiller grain, vitamins, minerals, mycotoxin sequestrant) (90% dry matter (DM), of which 18.5% is a crude protein (CP), 1.9 Mcal NEI/kg DM), 1 kg alfalfa hay (80% DM of which 18% CP, 1.35 Mcal/NEI/kg DM), and 1 kg corn silage (35% DM, 7% CP, 1.4 Mcal/NEI/kg DM). Water was allowed ad libitum. We fed the Holstein Friesian cows 5 kg alfalfa hay daily, 10 kg corn silage, and 5 kg corn concentrate. The cows had 2 to 4 births with 15 to 20 L of producing milk in the second third of lactation.

### 2.3. Crude Protein Extraction from Skimmed Goat and Cow Milk by Conventional Solvent Precipitation

Thirty milliliters of milk was skimmed by centrifugation at 4500× *g* for 30 min at 5 °C. We decanted to an approximate volume of 15 mL (of the 30 mL, only 15 mL was used). Then, 20 mL of ethyl alcohol was added and mixed, followed by the addition of 5 mL of chloroform and mixed at room temp. The mixture was incubated at −20 °C overnight and then centrifuged at 4500× *g* for 25 min at 5 °C. Then, we discarded the supernatant, and the pellet was allowed to stand to volatilize the solvent (to dry the pellet). We added 15 mL of 90% acetone at −20 °C for washing the pellet, and then the sample was mixed and incubated on ice for 15 min. Centrifugation continued at 4500× *g* for 25 min at 5 °C; the supernatant was discarded, and the washing procedure was repeated twice to dry the pellet. The protein pellet was suspended with 10 mL of HPLC water, and the proteins were quantified [17].

### 2.4. Total Protein Quantification by Fluorescence-Based Protein Assay

The concentration of proteins in milk was determined by Qubit^®^ 2.0 Fluorometer equipment. Three tubes for the protein standards and one tube for each sample were used for the quantification. The working solution was prepared by diluting the QubitTM reagent at 1:200 in the QubitTM buffer. Then, 200 μL of the working solution was prepared for each sample and standard. The samples were prepared according to the manufacturer’s instructions. The tubes were vortexed for 2 to 3 s, incubated for 2 min at room temperature for approximately 15 min, and protected from the dark. The tube was inserted in the Qubit 2.0 Fluorometer for quantification, and the reading was recorded [17].

### 2.5. Resolution of Proteins by Gradient SDS-PAGE

Separation of milk proteins was carried out by electrophoresis in 4–20% polyacrylamide gradient gel (SDS-PAGE) under reducing conditions. The identification of proteins was performed by comparing their relative mobility with 7 µL of known standard (Precision Plus Protein Dual Xtra Standards-BIO-RAD) by gel. For the quantitative analysis of the proteins of each milk sample, densitometric analysis was carried out using the Gel DOCTM EZ Imager equipment. The expression of proteins was reported in relative abundance.

### 2.6. Amino Acid Sequencing of BC Fraction by HPLC-MS/MS

Modified isoelectric precipitation extracted the whole casein fraction from goat and cow milk [18]. The milk samples were homogenized at 60 °C, and fat was separated by centrifugation at 2600× *g* for 20 min at room temperature. Then, we adjusted the milk pH to 4.6 by adding 10% (*v*/*v*) acetic acid while stirring at 30 °C. After isoelectric precipitation, samples were centrifuged at 2600× *g* for 20 min at room temperature, and whole casein pellets were washed twice in acidic distilled water (pH 4) under the same conditions. Complete casein pellets were resuspended in distilled water at pH 7.5. Protein concentration in whole casein extracts was determined as described above.

Whole casein samples were separated according to UREA-PAGE [19]. Protein samples were mixed 6:1 (*v*/*v*) with a 0.12 M Tris base, 2.5 mM EDTA, 8.2 M urea, 0.2 M 2-mercaptoethanol, and 0.01% (*w*/*v*) bromophenol blue 6× sample loading buffer. Working samples were heat-denatured at 95 °C for 5 min, and 7.9 μg of protein was loaded into a polyacrylamide gel consisting of 4% T, 4.5 M urea stacking gel pH 7.6 and 15% T, 9 M urea resolving gel, pH 8.9. Vertical electrophoresis was performed in a Mini PROTEAN Tetra Cell (Bio-Rad) using a 0.02 M tris base 0.19 M glycine running buffer pH 8.3. Separation was performed at four °C using 0.01 A for 15 min, followed by 0.03 A for two h. The gel was stained with Coomassie brilliant blue G-250 and documented on a GelDoc EZ system (Bio-Rad). We analyzed the image with Image Lab Software version 4.1 build 16, 22 Jun 2012 (Bio-Rad Laboratories, Ciudad de México, Mexico). As previously described, the amount of BC in whole casein samples submitted to amino acid sequencing was also determined by densitometric analysis.

Protein bands BC were excised from the gel, reduced with 1 mM DTT, alkylated with 5.5 mM iodoacetamide, and digested with thermolysin. The resulting peptides were desalted using Zip Tips C18 (Millipore, Billerica, MA, USA). We applied an EASY-nLC II nano-flow high-performance liquid chromatography system (Thermo-Fisher, San Jose, CA, USA) coupled to a-LTQ Orbitrap Velos ion trap mass spectrometer (Thermo Fisher) with a nano-electrospray ionization source (ESI) [20]. Nano-flow liquid chromatography was performed using a linear gradient of 10–80% (*v*/*v*) mobile phase (acetonitrile in 0.1% (*v*/*v*) formic acid aqueous solution) for 120 min with a house-made capillary column (0.75 μm ID × 10 cm L, RP-C18). The constant flow rate was used (300 nL/min).

For peptide fragmentation, we chose collision-induced dissociation (CID) and high-energy collision dissociation (HCD). All spectra were acquired in the positive ion mode. Fragmentation data collection was performed as a function of total ion scan according to predetermined amounts with an isolation width of 3.0 (m/z), normalized collision energy of 35 arbitrary units, an activation q = 0.250, an activation time of 10 ms, and a maximum time of injection of 10 ms. Data collection was performed with Proteome Discoverer™ Software (Thermo Fisher, San Jose, CA, USA). The amino acid sequence of BC A2 and BC A1 present in whole casein samples and the mean ± SEM (standard error of the mean) of their relative abundance were duplicated for each independent IF.

### 2.7. Statistical Analysis

Fractions of the total proteins in goat and cow milk were generally distributed as evaluated by Shapiro–Wilk (*p* > 0.05) and showed homogeneity of variance (Levene Statistic *p* > 0.05). One-way ANOVA was used to perform analysis of the total protein fractions in goat and cow milk, and significant difference (*p* < 0.05) among fractions was calculated using the Tukey test (SPSS software v. 22.0, Armonk, NY, USA).

## 3. Results

### 3.1. Protein Quantification

Table 1 shows the protein quantification results by Qubit^®^ 2.0 Fluorometer equipment. The protein concentration was necessary for the protein profile of goat milk and cow milk. The relative abundance of each protein was known by the total protein concentration in each goat and cow milk sample. Protein content ranged from 4.82 mg/mL in the Creole breed to 3.17 mg/mL in the French Alpine breed. No statistical difference was observed between different breeds.

### 3.2. Protein Profile of Goat Milk and Cow Milk

**Figure 1:**
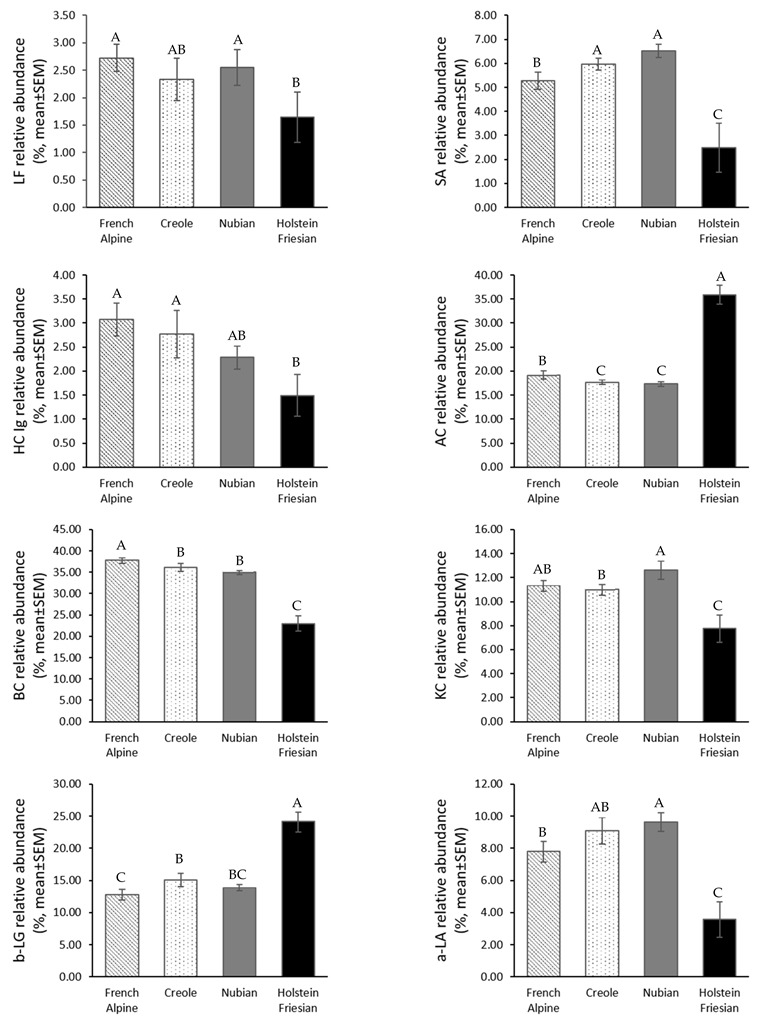
Mean relative abundance (%) ±SEM of proteins from French Alpine, Creole, and Nubian goat breeds, as well as from Holstein Friesian cows’ skimmed milk samples, resolved in 4–20% gradient polyacrylamide gel under denaturing and reducing conditions. Gels were stained with colloidal Coomassie blue G-250 and documented using the GelDoc EZ imager (Bio-Rad). Data were recorded and integrated as optical density from gel band peak areas using Image Lab 5.0 software (Bio-Rad). LF: lactoferrin, SA: seroalbumin, HC Ig: Heavy chain Immunoglobulin, AC: alpha-casein, BC: beta-casein, KC: kappa-casein, b-LG: beta-lactoglobulin, a-LA: alpha-lactoalbumin. A, B, and C indicate a significant statistical difference among breeds and species (*p* < 0.05).

Figure 1 compares the goat milk protein profile from each goat and Friesian cow breed. The protein profile was divided into caseins (AC, BC, and KC) and whey protein (LF, seroalbumin (SA), Ig, b-LG, and a-LA). Electrophoresis SDS-PAGE separated these proteins under reducing conditions.

The proteins that presented a lower abundance in goat milk than cow milk were AC. The protein AC was 17.67 ± 0.46 for the Creole breed, 19.18 ± 0.88 for French Alpine, 17.35 ± 0.49 for Nubian, and 35.92 ± 1.96 for Holstein cows. The relative abundance obtained from AC was statistically different with goats and cows, and this protein was vital because it is related to allergies, with a similar case for b-LG. After all, this protein is not present in human milk. The abundance of b-LG was 15.05 ± 1.02 for the Creole breed, 12.87 ± 0.82 for French Alpine, 13.96 ± 0.55 for Nubian, and statistically different for Holstein cows (24.15 ± 1.63).

Other proteins such as BC and KC were superior in goat milk compared to cow milk. These proteins have relevance in bioactive peptides generated by hydrolysis and cheese elaboration. Figure 1 also shows the protein relative abundance of BC for the Creole breed (36.13 ± 0.87), 37.82 ± 0.70 for French Alpine, 35.08 ± 0.48 for Nubian, and 22.96 ± 1.76 for Holstein cows. Where the French Alpine was different from others goat breeds and species, this is important because the separation method of the BC genetic variants and BC sequence in goat milk could be different.

### 3.3. Amino Acid Sequencing of BC Fraction by HPLC-MS/MS

Table 2 shows the peptides obtained after hydrolysis of goat milk from different breeds. The Creole breed has a peptide not identified in Alpine French or Nubian breeds. The amino acid sequence of BC from goat milk from three breeds included in the study showed that the amino acid present at position 67 is Proline; in 66, it corresponds to Isoleucine. The difference in amino acid 67 relates to the BC type and the sequence of BCM-7. Additionally, the BC from different samples studied here showed the presence of a Valine–Proline–Proline (VPP) tripeptide, which acted as an inhibitor of the angiotensin-converting enzyme (ACE).

## 4. Discussion

### 4.1. Goat Milk and Cow Milk Protein Profile

In the protein profile, the relative abundance of AC in goat milk, regardless of the breed, was statistically significant in cow milk (*p* < 0.05). The lower levels of AC in goat milk are likely related to the lower allergenicity of goat milk compared to cow milk, which is a key benefit for infants using goat milk as their main source of nutrients and for individuals suffering from allergies to cow milk [20]. For the BC, the relative abundance was more significant in goat milk than in cow milk (Figure 1). The difference in the BC content among goat breeds was statistically significant.

There were significant differences (*p* < 0.05) in the content of KC between goat milk and cow milk. There was a considerable difference in KC between the Creole and the Nubian breed, as is reported in Figure 1. KC protein is associated with the elaboration and consistency of curdles, and the difference between species affects the manufacturing of the cheese [21,22].

SA binds free fatty acids participating in the synthesis of lipids. Its antioxidant activity has been reported to protect lipids against phenolic-induced oxidation [23]. The relative abundance of SA protein shows a statistical difference between the French Alpine (5.28 ± 0.36) and Nubia; the highest percentage is for the Nubian breed (6.51 ± 0.28). The content of SA is more elevated in goat milk than in cow milk. This is important for the antioxidant character of goat milk.

The relative abundance of LF for the goat breeds was not significantly different, but the Alpine and Nubian milk differed significantly from cow milk. The LF is a globular multifunctional protein that binds, transports, and supplies the organism with iron. The iron-binding properties seem to vary between LF from different species [24]. Furthermore, it has been recognized as having antimicrobial and antifungal properties [25]. Research has revealed that bovine LF induced apoptosis in human stomach cancer cells [26].

Concerning the b-LG protein, its relative abundance is similar in the Creole (15.05 ± 1.02%) and Nubian (13.96 ± 0.53%) goat milk, while the Alpine French showed the lowest close lot for this protein (12.78 ± 0.82%). The relative abundance of b-LG in goat breeds was significantly different from that of cow milk. B-LG is the most significant component of whey proteins in cow milk, contributing 20% of the total protein. The homology of b-LG tertiary structure with the plasma retinol-binding protein, as well as its high stability against the proteolytic action of digestive enzymes, has suggested the role of this protein as a resistant carrier of retinol. Moreover, fatty acid-binding sites characterized in b-LG allow this protein to participate in the digestion of milk lipids during the neonatal period. In addition, it has been shown that b-LG enhances intestinal uptake of retinol, triglycerides, and long-chain fatty acids in pre-ruminant calves [27], and it has been speculated that this protein may play a role in the absorption and subsequent metabolism of fatty acids. Other possible functions have been described for this whey protein, such as its role in developing passive immunity with IgG [28]. B-LG is a rich source of Cys, an essential amino acid that appears to stimulate glutathione synthesis, an anticarcinogenic tripeptide produced by the liver for protection against intestinal tumors [29]. The high nutritional and functional value of b-LG is widely recognized and has made this protein an ingredient of choice in the formulation of modern foods and beverages [30].

The relative abundance of a-LA in goat milk was 9.65 ± 0.58% for Nubian, 7.81 ± 0.67% for French Alpine, and 9.08 ± 0.83 for Creole. There were significant differences among the Alpine and Nubian breeds. The relative abundance of a-LA in cow milk (3.58 ± 1.13) was significantly different (*p* < 0.05) for goat breeds, with a lower ratio than that of goat milk. In human milk, it is the most predominant whey protein, constituting 10–20% of the total protein in mature milk [31]. Some important bioactivities, the best known being the anti-tumoral activity and antimicrobial activity [32], were observed for the complex between human a-LA and oleic acid, called HAMLET (human alpha-lactalbumin made lethal to tumor cells). This complex can kill tumor cells by a process resembling programmed cell death. HAMLET has broad antitumor activity in vitro in a human glioblastoma rat xenograft model in patients with skin papillomas and bladder cancer [33]. Likewise, a complex of bovine a-LA and oleic acid (BAMLET) killed tumor cells via a mechanism involving lysosomal membrane permeabilization, showing potent cytotoxic activity against eight cancer cell lines [34].

### 4.2. Goat Milk BC

The commercial proteolytic enzyme thermolysin has released several bioactive peptides from caseins and whey proteins [35]. Table 2 shows the peptide sequences found when the hydrolyzed BC protein from goat milk used the thermolysin enzyme. Like human milk, goat milk contains bioactive peptides, nucleotides, polyamines, sialic acid, free amino acids, and growth factors optimizing human development. Goat milk antimicrobial activity is attributed to immunoglobulins and immuno-proteins such as lactoferrin, lactoperoxidase, and lysozyme. The antibacterial peptides are active agonists against pathogenic microorganisms such as Escherichia, Helicobacter, Listeria, Salmonella, Staphylococcus, yeasts, and filamentous fungi [36].

Chessa et al., 2009 [37], found goat milk bioactive peptides related to cow milk, and of these, nine showed opioid activity, five were protein-binding, twenty-three showed hypotensive activity, three were antithrombotic, and 6 functioned as immunomodulators. BCM−7 is a peptide released by the hydrolytic action of BC, which is associated with an increased risk of diseases such as autism, cardiovascular disease, and type I diabetes [38]. Protein genetic variants determine its release and are associated with the specific breed. The main polymorphic variants of milk BC from the most common dairy cattle species are A1, A2, and B. The amino acid present at position 67 of the BC sequence is critical for the release of BCM-7. Invariant A2, a Proline residue, occurs at position 67, wherein variants A1 and B have a histidine residue. In the case of the A2 variant, hydrolysis of Isoleucine 66-Proline 67 does not occur, or it is performed at a low range. For variant A1, the release of BCM-7 is generated by the action of pepsin. Proteolytic synthesis is involved in the fermentation of milk or in the manufacturing of cheese, and it can potentially hydrolyze BC to BCM-7 and other beta-casomorphins [39]. Results from the hydrolysis process and the sequencing of the analyzed peptides showed that for the BC from three goat breeds, the amino acid present in position 67 is a Proline, and in position 66, it corresponds to Isoleucine, so it is assumed that the BC variant of goat milk is an A2-type so that in its hydrolytic process, the peptide BCM-7 is not produced or is produced in low amounts. It has great relevance for human nutrition and is different from cow milk.

Other peptides released in BC hydrolysis are tripeptides inhibiting the angiotensin-converting enzyme (ACE), which has the following amino acid sequence: Valine–Proline–Proline (VPP) and Isoleucine–Proline–Proline (IPP), and they are found in goat milk or derived products such as cheese, which show cardiovascular and antihypertensive activity [40]. These peptides are enormously increased by the presence of tripeptide hydrophobic chains with aromatic or branched amino acids [40]. Pihlanto-Leppälä et al., 1998 [41], demonstrated that the fermentation of milk with a cell crop is not enough to generate the inhibitory activity of ACE and that digestion with pepsin and trypsin is necessary. This explains what can happen under in vivo conditions in the gastrointestinal tract when fermented products are ingested. In the Holstein cow milk BC, two precursor amino acid sequences of ACE-inhibiting peptides are in positions 74 and 84. In contrast, in goat milk BC, they only have a precursor sequence of said peptides at position 84.

## 5. Conclusions

In the present work, it is reported that in goat milk, the concentration of AC is much lower than that of BC, which has an important implication for human nutrition since it is a protein (AC) with high allergenicity. The breeds of goats have higher a-LA than cows; therefore, goat milk is considered an alternative food with less allergenicity than cow milk and protein of higher biological value (a-LA). The amino acid present in position 67 of BC from three goat breeds is a Proline, so it is assumed that the BC variant of goat milk is an A2 type. Therefore, in its hydrolytic process, the peptide BCM-7 is not produced or is produced in low amounts. It has great relevance for human nutrition and is different from cow milk. The hydrolyzed peptides of BC among breed goats are different in quantity; this is important in terms of their biological activity in the human body.

## Figures and Tables

**Table 1:** Total protein concentrations determined in skimmed milk samples obtained from French Alpine, Creole, and Nubian goat breeds, as well as from Holstein Friesian cows.

Sample	Total Protein (mg/mL)
Creole	4.82 ± 0.80
French Alpine	3.17 ± 0.71
Nubian	3.57 ± 0.69
Cow (Holstein)	3.42 ± 0.44

Mean ± S.D, *n* = 10 animals by breed and species.

**Table 2:** Goat milk BC hydrolyzed peptide sequences.

Protein: Beta-Casein Gene: CSN2		
Bos Taurus (Bovine) Molecular weight: 24 KDa Signal peptide: 15 amino acids Mature chain: 209 amino acids	Capra Hircus (Caprine) Molecular weight: 23.3 KDa Signal peptide: 9 amino acids Mature chain: 207 amino acids	
Amino acid sequence 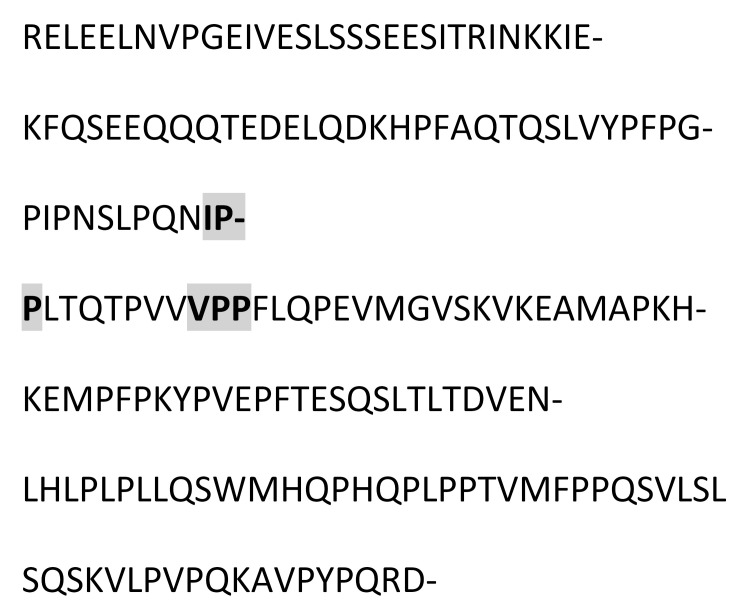	Amino acid sequence 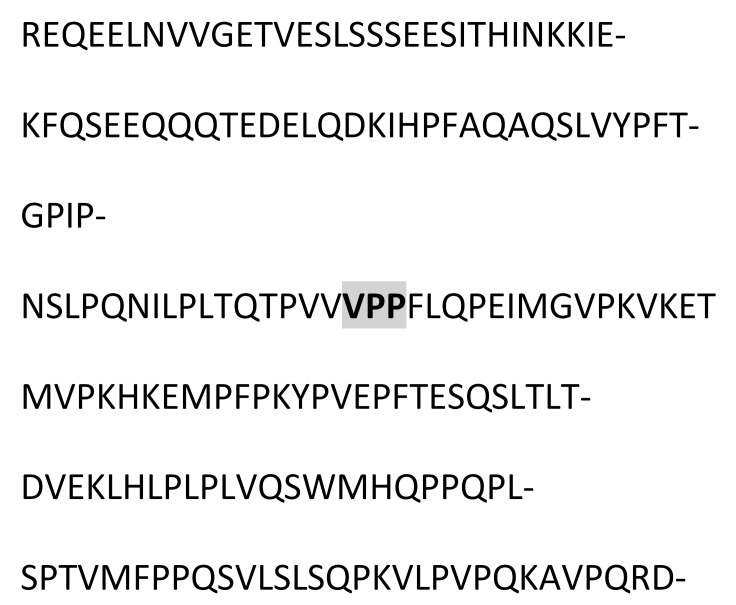	Difference in 43 Amino acid
**Breed of goats**		
Alpine French sequence	Nubian sequence	Creole sequence
* 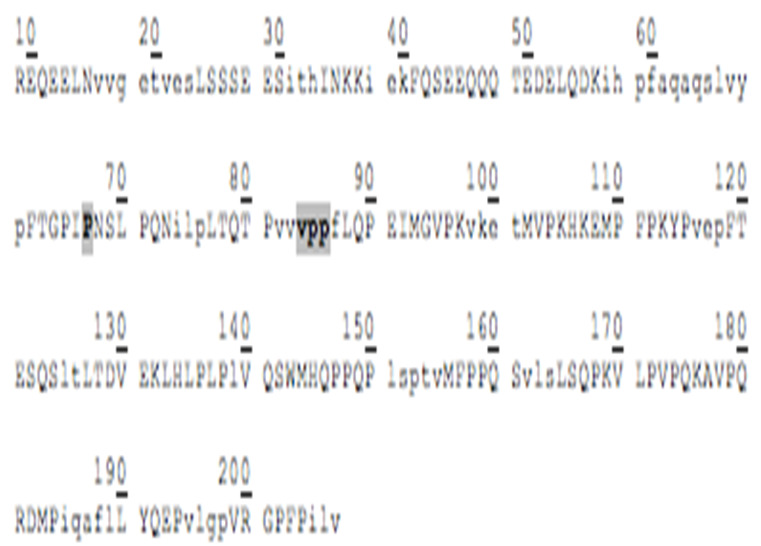 *	* 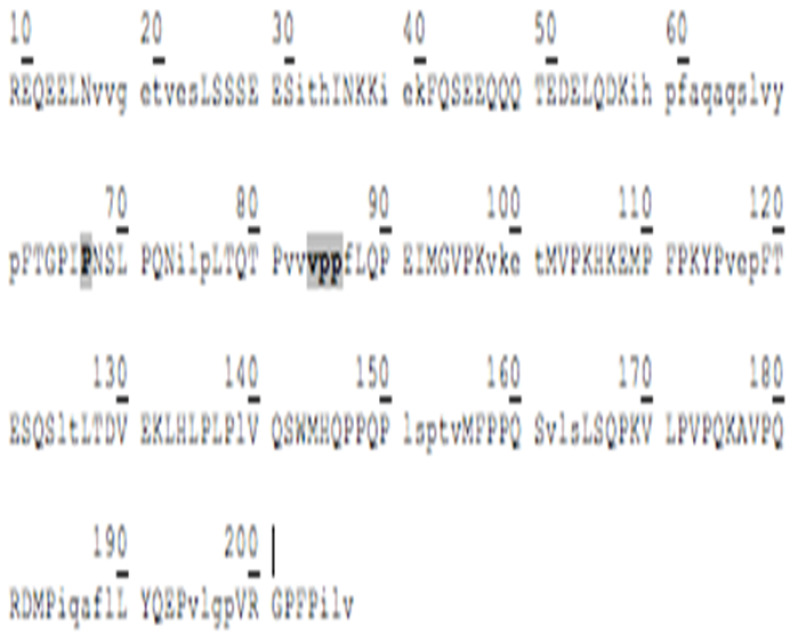 *	* 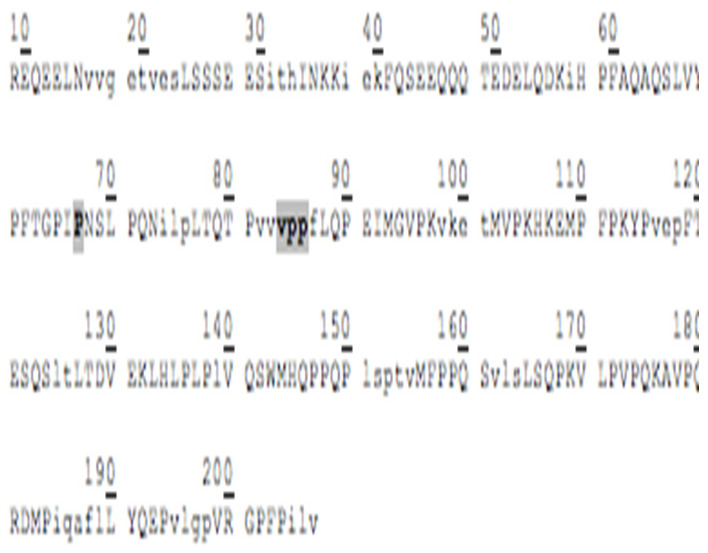 *
hydrolyzed peptides (20)	hydrolyzed peptides (20)	hydrolyzed peptides (21)
FQSEEQQQTEDELQDK	FQSEEQQQTEDELQDK	FQSEEQQQTEDELQDK
mVPKHKEmPFPKYP	mVPKHKEmPFPKYP	mVPKHKEmPFPKYP
------------------	------------------	HPFAQAQSLVYP
FTGPIPNSLPQN	FTGPIPNSLPQN	FTGPIPNSLPQN
REQEELN	REQEELN	REQEELN
AVPQRDmP	AVPQRDmP	AVPQRDmP
mHQPPQP	mHQPPQP	mHQPPQP
VLPVPQK	VLPVPQK	VLPVPQK
LSSSEES	LSSSEES	LSSSEES
mFPPQS	mFPPQS	mFPPQS
LTDVEK	LTDVEK	LTDVEK
FTESQS	FTESQS	FTESQS
LHLPLP	LHLPLP	LHLPLP
VRGPFP	VRGPFP	VRGPFP
LYQEP	LYQEP	LYQEP
LQPEI	LQPEI	LQPEI
LSQPK	LSQPK	LSQPK
LTQTP	LTQTP	LTQTP
mGVPK	mGVPK	mGVPK
VQSW	VQSW	VQSW
INKK	INKK	INKK

P Proline amino acid at position 67. IPP, VPP Peptides released in the BC hydrolysis. ------------------, The peptide is not present.

## Data Availability

The data presented in this study are openly available in the repository institutional DGBSDI-UAQ at http://ri-ng.uaq.mx/handle/123456789/807 (accessed on 12 October 2015), reference number (RI007450).
